# Influence of season and sex on hemato-biochemical traits in adult turkeys under arid tropical environment

**DOI:** 10.14202/vetworld.2016.530-534

**Published:** 2016-05-28

**Authors:** Anil Gattani, Arti Pathak, Ajeet Kumar, Vaibhav Mishra, Jitendra Singh Bhatia

**Affiliations:** 1 Department of Veterinary Biochemistry, Bihar Veterinary College, Patna, Bihar, India; 2 Department of Veterinary Physiology & Biochemistry, Apollo College of Veterinary Medicine, Jaipur, Rajasthan, India

**Keywords:** alanine aminotransferase, aspartate aminotransferase, season, sex, turkey

## Abstract

**Aim:**

The objective of this study was to evaluate the effect of season and sex on hemato-biochemical parameters of turkey (*Meleagris gallopavo*) in the arid tropical environment.

**Materials and Methods:**

The experiment was conducted on 20-week old turkeys consisting of 20 males and 20 females. Blood was collected from all turkeys during January and May. Hemoglobin (Hb), red blood cell (RBC), packed cell volume (PCV), mean corpuscular volume (MCV), mean corpuscular hemoglobin (MCH), and mean corpuscular hemoglobin concentration (MCHC) were estimated in whole blood and glucose, protein, albumin, globulin, A/G ratio, calcium, phosphorus, alanine aminotransferase (ALT), and aspartate aminotransferase (AST) in serum.

**Result:**

Season has significant (p<0.05) effect on Hb concentration, RBC, and PCV in both male and female. Male has significantly higher (p<0.05) Hb concentration, RBC, and PCV. There is no significant effect of sex, and season was observed on MCV, MCH, and MCHC. Glucose, protein, albumin, globulin, and A/G ratio were significantly (p<0.05) affected by season and sex. AST and ALT were significantly (p<0.05) affected by season in both sexes. There is no significant difference was recorded on calcium, phosphorus due to season and sex.

**Conclusion:**

Under arid tropical environment, turkey hemato-biochemical parameters are influenced by both sex and season.

## Introduction

Turkey (*Meleagris gallopavo*) is a prized avian species reared all over the world for their tasty and high-quality meat besides, its link with celebrations of “Christmas” and “Thanksgiving celebrations” in the western world [1]. It originates from North America and has now been introduced nearly worldwide including India. The production of this poultry species is gaining momentum as a new agricultural activity for the commercial production of meat in India as a source of animal protein due to its comparatively high percentage of protein and low percentage of fat [2-4]. In general, blood examination is considered most dependable indicator of health status. Blood biochemical profile such as glucose, calcium, total protein, aspartate aminotransferase (AST), alanine aminotransferase (ALT), urea, and chloride levels are of diagnostic values for various disease conditions and having particular reference to liver disorders, kidney diseases, diarrhea, dehydration, etc. [5]. Enzyme activity can be useful in selecting males to improve fertility and or hatchability of females in chicken.

The information on these is useful for diagnostic and management purposes which could further be incorporated into breeding programs for genetic improvement of turkeys. Values for the hematology and serum biochemical characters for turkey have been reported elsewhere [6,7], but literature is still incomplete with respect to variability of these parameters in the different climatic seasons, especially in the semi-arid region of India. Thus, to generate baseline data, the present study was planned. This will help in furtherance of research on breed improvement programs at geo-climatic conditions of this tract.

Therefore, the present study was undertaken to evaluate the effect of sex and season on the hematology and serum biochemical profile of turkey in the arid tropical environment.

## Materials and Methods

### Ethical approval

The prior approval from the Institutional Animal Ethics Committee (Apollo College of Veterinary Medicine, Jaipur) was obtained for use of animal in this study.

### Experimental animals

The present experiment was conducted at Poultry complex, Apollo College of Veterinary Medicine, Jaipur, from January to May 2011 on 20 weeks old Beltsville small white turkey. During the experiment, in January 2011, average temperature was 6.8°C (1.4-13.8) and mean humidity was 54.6%, and in May 2011, average temperature was 34.4° (39.6-13.9) and mean humidity was 27.4%. A total of 40 adult turkeys (20 males and 20 females) were selected for this study. The turkeys were housed in a floor pen and were fed standard chicken layer ration supplied by Godrej agrovet. Feed and water provided *ad lib*. Deworming of birds was done regularly.

### Collection and analysis of blood samples

Blood samples were collected aseptically two times in summer and winter season from the superficial ulnar vein of all 40 turkeys in EDTA containing vial for hematological study. Hematological study was performed within 24 h after blood collection. Hemoglobin (Hb) was estimated using drabkin’s solution (Span diagnostic, India); red blood cell (RBC) using Neubauer chamber; other erythrocytes indices such as mean corpuscular volume (MCV), mean corpuscular hemoglobin (MCH), and mean corpuscular hemoglobin concentration (MCHC) were estimated using the standard formulae. Blood samples were also collected for separation of serum and stored at −20° for further use. These serum samples were used for biochemical studies. The enzymatic estimations were done within 24 h of collection. The serum biochemical parameters including serum glucose, protein, albumin, globulin, A/G ratio, calcium, phosphorus, ALT, and AST were estimated using kits (Span diagnostic, India).

### Statistical analysis

All the data were expressed as mean±standard error (SE) values. The data were subjected to statistical analysis by Paired Sample t-test using SPSS version 17 according to Snedecor and Cochran [8].

## Results

The results of the hemato-biochemical parameters of turkey in different season and sex are summarized as mean±SE in Table-1.

**Table-1:** Effect of season and sex on hemato-biochemical profile in Turkey (mean±SE).

Hemato-biochemical attributes	Sex	Season	

		Winter	Summer
Hemoglobin (g/dl)	Male (20)	11.97^B^±0.25	10.88^bA^±0.25
	Female (20)	11.43^B^±0.36	10.09^aA^±0.22
PCV (%)	Male (20)	40.95^bB^±0.78	34.55^bA^±0.54
	Female (20)	38.85^aB^±0.65	32.65^aA^±0.70
RBC (millions/mm^3^ of blood)	Male (20)	2.97^bB^±0.15	2.65^bA^±0.07
	Female (20)	2.55^aB^±0.10	2.33^aA^±0.08
MCV (fl/cell)	Male (20)	144.35±7.52	131.88±3.91
	Female (20)	157.44±7.27	141.76±4.48
MCH (pg/cell)	Male (20)	42.57±2.64	41.67±1.46
	Female (20)	45.86±2.02	43.88±1.44
MCHC (g%)	Male (20)	29.42±0.79	31.60±0.70
	Female (20)	29.55±1.03	31.16±0.89
Glucose (mg/dl)	Male (20)	251.61^bB^±12.19	220.42^bA^±8.9
	Female (20)	219.49^aB^±8.45	193.53^aA^±7.34
Total protein (g/dl)	Male (20)	4.12^aB^±0.15	3.42^aA^±0.09
	Female (20)	5.45^bB^±0.16	4.25^bA^±0.25
Albumin (g/dl)	Male (20)	1.45^aB^±0.03	1.31^aA^±0.04
	Female (20)	1.62^bB^±0.04	1.45^bA^±0.04
Globulin (g/dl)	Male (20)	2.66^aB^±0.15	2.10^aA^±0.08
	Female (20)	3.83^bB^±0.17	2.79^bA^±0.25
A/G ratio	Male (20)	0.58^b^±0.03	0.64±0.03
	Female (20)	0.44^aA^±0.03	0.59^B^±0.04
ALT (IU/L)	Male (20)	14.45^A^±1.86	32.07^B^±1.86
	Female (20)	15.47^A^±1.67	29.84^B^±2.32
AST (IU/L)	Male (20)	309.22^A^±7.95	348.35^B^±9.20
	Female (20)	282.99^A^±15.64	333.52^B^±8.39
Ca (mg/dl)	Male (20)	7.82±0.28	8.04±0.20
	Female (20)	7.85±0.39	8.46±0.22
P (mg/dl)	Male (20)	5.31±0.33	4.82±0.28
	Female (20)	4.74±0.28	4.66±0.24

Superscripts (A, B) indicate significant difference (p<0.05) row wise. Different superscripts (a, b) indicate significant difference (p<0.05) column wise. SE=Standard error, PCV=Packed cell volume, RBC=Red blood cell, MCV=Mean corpuscular volume, MCH=Mean corpuscular hemoglobin, MCHC=Mean corpuscular hemoglobin concentration, ALT=Alanine aminotransferase, AST=Aspartate aminotransferase

### Hematological profile

The erythrocytic profile is represented in Table-1. Hb in male was 11.97±0.25 and 10.88±0.25 during winter and summer season, respectively, whereas in the female, it was 11.43±0.36 and 10.09±0.22 in both the seasons. Hb is significantly (p<0.05) affected by the season in both the sexes. Significant (p<0.05) effect of sex and season was observed on packed cell volume (PCV) and RBC. Significantly (p<0.05) lower PCV values were observed in female (38.85±0.65 and 32.65±0.70), in both winter and summer seasons, than male (40.95±0.78 and 34.55±0.54). However, both male and female have significantly (p<0.05) higher value during the winter season than summer. Similarly, RBC is significantly (p<0.05) higher in male (2.97±0.15 and 2.64±0.07) than female (2.55±0.10 and 2.33±0.08), in winter and summer season, respectively. MCV and MCH were marginally higher during the winter season in both sexes. Further female has higher MCV and MCH than its male counterpart. MCHC was slightly higher during the summer season in male (31.60±0.70) and female (31.16±0.89) than winter season (29.42±0.79 and 29.55±1.03), respectively.

### Biochemical profile

Male has significantly (p<0.05) higher glucose level in comparison to female. In the winter season, the level of glucose was significantly (p<0.05) higher both in male (251±12.19) and female (219.49±8.45) than that of summer season (220.42±8.92 and 193.53±7.34, respectively). Total protein concentration was significantly (p<0.05) higher in female during winter and summer season (5.45±0.16 and 4.25±0.25) than their male counterpart (4.12±0.15 and 3.42±0.09). Further, both in male and female the total protein level is significantly (p<0.05) higher in the winter season. Albumin concentration in serum was significantly (p<0.05) influenced by the season both in male and female. Female had significantly (p<0.05) high concentration (1.62±0.04 and 1.45±0.04) of albumin than male (1.45±0.03 and 1.31±0.04) in winter and summer season, respectively. Significantly (p<0.05) high globulin concentration was observed in female during both the summer and winter season (3.83±0.17 and 2.79±0.25) than male (2.66±0.15 and 2.10±0.08). Furthermore, in winter season, globulin was significantly (p<0.05) higher than summer season in both sexes. The climatic season had a significant (p<0.05) effect on A/G ratio in female. Further, male (0.58±0.03) has significantly (p<0.05) high value than female (0.44±0.03) only in winter season. ALT and AST enzyme was studied in the present study, and both have the similar trend in respect to season. In the summer season, the activity of ALT and AST was significantly (p<0.05) increased. In male, ALT activity was 15.47±1.67 and 32.07±1.86 during winter and summer season, respectively, whereas in the female, it was 14.45±1.86 and 29.84±2.32. Similarly, the AST activity was 309.22±7.95 and 348.35±9.20 in male and 282.99±15.64 and 333.52±8.39 in female during winter and summer season, respectively.

The serum concentration of Ca is non-significantly higher in the summer season. Further, female has non-significantly higher concentration (7.85±0.39 and 8.46±0.22) than male (7.82±0.28 and 8.04±0.20) during winter and summer season. A reverse trend was observed for phosphorus concentration in serum. Male has non-significantly high phosphorus (5.31±0.33 and 4.82±0.28) than female (4.74±0.28 and 4.66±0.24) in their blood during winter and summer season, respectively.

## Discussion

Hematological and serums’ biochemical parameters can provide importance information for animal’s immune status and beside of diagnostic and management purposes, can be used for developing new strains that are genetically resistant to poultry diseases as well as for genetic improvement programs [9].

### Hematological traits

The erythrocytic profile in the present study is in the range reported by other workers in different part of the globe [10,11]. Higher values of Hb, PCV, and RBC observed during winter months in both male and female turkey is in accord with finding in Nigerian ducks [12]. The variation in the environment temperature and photoperiod is considered as the most important factor that affects the erythrocyte count, Hb, and hematocrit values. A decline in environmental temperature resulted in significant alterations of the circulatory system. This might be due to low ambient temperature result in high oxygen demand by the body, low partial pressure of oxygen in the blood (hypoxemia), and higher metabolic rate (favors high feed intake), which stimulate erythropoiesis thereby producing higher hematological values in winter [13,14]. During summer, high ambient temperature increases body temperature, respiration and respiratory water loss and oxygen consumption of birds. The increased oxygen intake increases the partial pressure of oxygen in the blood of birds [15] leading to decreased erythropoiesis and consequently, reducing the number of circulating erythrocyte [16].

MCV, MCH, and MCHC did not show any statistical differences between seasons, whereas numerically lower values were obtained during summer season might be due to in changes in blood volume and blood viscosity [17]. Female turkeys show lower Hb, PCV, and TEC in comparison to male, might be due high estrogen concentration [17-19].

### Biochemical traits

The lowered blood glucose in female due to estradiol effect which decreases the expression of gluconeogenic genes in the liver [20,21]. Protein concentrations in the present study are in the reference range as reported elsewhere [20]. The higher protein concentration in females could be explained by the high level of estrogen hormones in females responsible of the high content of serum globulins [22]. The decrease in serum albumin during the summer season may be due to reduced protein consumption and consequently decreased supply of essential amino acids from feed, accompanied with reduced protein digestibility because exposure of broilers to high environmental temperature [23,24]. The mean activity of ALT and AST is in accordance with the other researchers [6,7,20]. The increased values of enzyme activity during summer season may be attributed to the greater influence of thermal stress [25].

Non-significant higher calcium level in female might be result of estrogen response [26,27]. Non-significant variation in serum phosphorus levels between sex can be attributed to hormonal influence and breeding activity [27]. Factors such as breed, dietary calcium source, housing system, and interaction between them affected the serum inorganic phosphorus values [28].

## Conclusion

The hemato-biochemical values of adult turkeys were determined under arid tropical environment for studying the effect of season and sex. The result found that season had a significant effect on Hb, RBC, PCV, glucose, protein, albumin, globulin, A/G ratio, ALT, and AST. The sex and season have no significant effect on MCV, MCH, MCHC, Ca, and P. The effect of season and sex should, therefore, be considered when interpreting the parameters to ensure accuracy and to avoid undesirable sources of variation and thus misjudgment for hemato-biochemical parameters under tropical environment.

## Authors’ Contributions

JSB and AG designed the experiment. AG, AP, and VM conducted the experiment. AG and AK did technical writing and revision of the manuscript. All authors have read and approved the final version of the manuscript.
